# Chondrosarcoma of the Mobile Spine and Sacrum

**DOI:** 10.1155/2011/274281

**Published:** 2011-03-03

**Authors:** Ryan M. Stuckey, Rex A. W. Marco

**Affiliations:** Department of Orthopaedics, University of Texas Medical School at Houston, 6700 West Loop South, Suite 110, Bellaire, TX 77401, USA

## Abstract

Chondrosarcoma is a rare malignant tumor of bone. This family of tumors can be primary malignant tumors or a secondary malignant transformation of an underlying benign cartilage tumor. Pain is often the initial presenting complaint when chondrosarcoma involves the spine. In the mobile spine, chondrosarcoma commonly presents within the vertebral body and shows a predilection for the thoracic spine. Due to the resistance of chondrosarcoma to both radiation and chemotherapy, treatment is focused on surgery. With en bloc excision of chondrosarcoma of the mobile spine and sacrum patients can have local recurrence rates as low as 20%.

## 1. Introduction

Chondrosarcoma is a rare malignant bone tumor that produces cartilage matrix. Chondrosarcoma can be subclassified as a primary malignant bone tumor or a secondary malignant transformation of an underlying enchondroma or osteochondroma. The estimated annual incidence has been reported as 1 in 200,000 [1]. The prevalence of chondrosarcoma in the mobile spine is reported between 6.5 and 10%, while approximately 5% are located within the sacrum [2–4]. Chondrosarcoma typically presents in patients between the 3rd and 7th decades.

## 2. Clinical Presentation

Patients with chondrosarcoma of the spine generally present with pain in the area of the lesion. The pain is often insidious in nature and can be present for weeks to years. Boriani et al. noted a mass at presentation in 34% of patients in their series [2], and Shives et al. reported 40% of patients with a mass at presentation [3]. The neurologic presentation can be somewhat mixed and can range from radicular pain to frank weakness. Radicular symptoms are seen in roughly 24% of patients [2, 5]. 

## 3. Location

Chondrosarcoma can occur within all regions of the spine. Roughly 20% of chondrosarcoma arises in the cervical spine, 30% in the thoracic spine, 20% in the lumbar spine, and 20% in the sacrum. Several series have shown a higher prevalence of chondrosarcoma in the thoracic spine. In the MD Anderson experience, 48% (10/21), 33% (7/21), and 19% (4/21) were located in the thoracic, lumbar, and cervical spines, respectively [5]. A similar breakdown was noted in the series reported by Shives et al. [3]. Bergh et al. reported on a series of 69 consecutive chondrosarcoma and found that 16% (11/69) were located in the sacrum [6]. 

**Figure 1:**
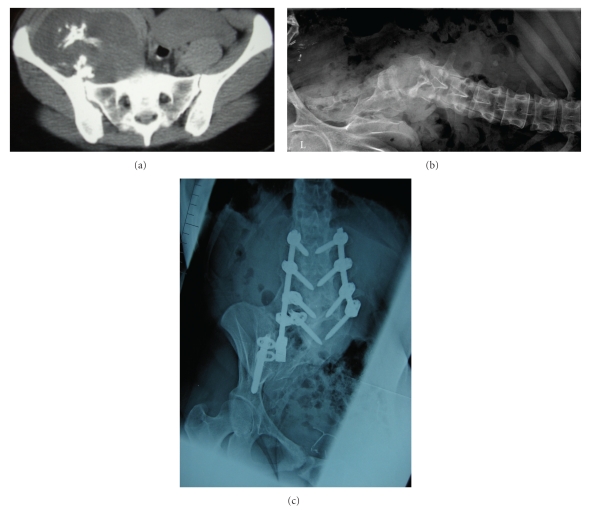
A 27-year-old female with a right sacroiliac secondary chondrosarcoma arising from a sacroiliac osteochondroma. The tumor involved half of the sacrum, and a sagittal resection through the midline of the sacrum was required to obtain wide margins. She developed scoliosis and sitting imbalance and underwent a subsequent posterior spinal fusion and instrumentation. She remains free of disease 48 months after surgery.

Within the vertebra, Chondrosarcoma can be isolated to the body, the posterior elements, or a combination of both. Primary chondrosarcoma is predominately located in the vertebral body, described as zones 10-3 according to the WBB staging system [2, 3]. In contrast, chondrosarcoma that arise from an underlying benign chondral lesion are typically located in the posterior elements of the vertebra. Sacral chondrosarcoma are typically located eccentrically in the upper portion of the sacrum (Figures 1(a), 1(b), and 1(c)). This location can lead to local involvement of the sacroiliac joints [7]. 

## 4. Staging

The appropriate staging of chondrosarcoma of the spine is essential for prognosis and surgical treatment. Appropriate staging includes radiologic and histologic information. Imaging includes plain radiographs, CT scan, and MRI to evaluate the tumor. Chondrosarcoma presents radiographically as a mixed lytic and blastic lesion. Low-grade lesions have dense spicules of calcification and an eccentric, lobular appearance. High-grade lesions can have amorphous areas of calcifications and concentric growth of a soft tissue component [8]. 

Additional imaging studies include a CT scan of the chest, abdomen, and pelvis, as well as an MRI of the spine to evaluate the rest of the spinal column. The appropriate imaging studies allow for a better understanding of the extent of the lesion as well as a differential diagnosis. 

The histologic diagnosis is essential to the staging and ultimate treatment of the spinal tumor. Prior excisional biopsies can adversely affect the treatment and survival of patients with chondrosarcoma of the spine [9]. Thus a closed, image-guided core needle biopsy with a trocar provides a safe and effective method of obtaining a histological diagnosis in patients with suspected chondrosarcoma of the spine. Histologic characteristics of chondrosarcoma include chondroid matrix, mitotic figures, hypercellularity, nuclear atypia, double-nucleated cells, and myxoid changes in the stroma [3, 4, 8, 10].

Two systems are commonly used to stage primary tumors of the spine. Enneking developed a system based on the location of the tumor, the histologic grade of the lesion, and whether metastases are present or not [11]. Low-grade tumors are designated with the Roman numeral (I) and high-grade lesions (II); if metastases are present, the tumor is designated by the Roman numeral (III). In addition the tumor is described as intracompartmental (A) or extracompartmental (B). This classification system was originally developed for tumors involving the appendicular skeleton and does have some limitations in the mobile spine. Weinstein (WBB) proposed a classification and surgical staging system developed specifically for spinal tumors [12]. The WBB staging system divides the vertebra into zones in the axial plane in a radiating pattern similar to a clock face. The numbers one through twelve designate the zones. The second portion of this system classifies the tumor based on layers designated by the letters A–E where (A) is prevertebral extraosseous, (B) intraosseous superficial, (C) intraosseous deep, (D) extraosseous extradural, and (E) extraosseous intradural. The WBB staging system then allows for direction in the method of resection. If the tumor involves zones 4–8 or 5–9, a vertebrectomy with a double approach is recommended, if zones 2–5 or 7–11 are involved, then a sagittal resection is recommended, and if the tumor involves zones 10-3, then a posterior arch resection is recommended.

## 5. Treatment

Chondrosarcoma are a family of slow-growing neoplasms. It has been well established in the literature that these tumors are resistant to chemotherapy and radiation therapy and thus Chondrosarcoma is a surgical disease [2–7, 10, 13]. En bloc or radical resection of chondrosarcoma involving the long bones has led to 5-year survival rates as high as 54 to 78% [4, 6, 10]. 

Surgical treatment consisting of either curettage or en bloc resection has been described for both primary and recurrent tumors [7, 13–16]. A description of the surgical margin is important for comparing results and the prognosis following surgical treatment. Intralesional excision refers to tumor cells present on the surface of the resected tissue. Marginal excision refers to a thin layer of tissue that is reactive, but without neoplastic tissue. Wide excision refers to a tumor-free cuff of normal tissue surrounding the lesion. 

In the mobile spine and sacrum, the inherent intimate relationship of the neurovascular structures and need for structural stabilization can make en bloc resection difficult, and in some cases intralesional curettage is more appropriate. Boriani et al. proposed a set of criteria directing treatment toward curretage. These criteria include circumferential spinal canal involvement, the need for spinal cord ligation to complete en bloc resection, and the potential for spinal cord ischemia from ligation of the spinal segmental artery [2]. 

Unfortunately, results following curretage are poor. In their series of twenty-two patients with chondrosarcoma of the mobile spine, Boriani et al. reported at least one local recurrence or progression of disease in 100% of patients (10/10) treated with curretage. 80% of these patients died at a mean of thirty-six months [2]. In contrast, 25% (3/12) of patients treated with en bloc marginal resection had a local recurrence. The margins were classified as contaminated or intralesional in two of the three patients with local recurrence after en bloc excision. Shives et al. reported on twenty patients with chondrosarcoma of the mobile spine. Similar to the previously cited study, 100% (11/11) of patients treated with intralesional excision had documented disease progression at a mean of 24.8 months. All of the patients treated with an intralesional excision died [3]. York et al. found a 69% recurrence rate in patients treated intralesionally with a subtotal excision at a mean of 44.4 months compared to a 20% recurrence rate in those treated with en bloc resection [5].

The role of en bloc resection in the treatment of chondrosarcoma of the sacrum has also been well established [7, 13]. Fourney et al. reported on three patients with chondrosarcoma in their series on en bloc resection of sacral tumors. All three patients had local recurrence, but it is important to note that all three patients had previously undergone a subtotal excision prior to their planned en bloc resection [7]. This further supports the important role of negative margins and en bloc resection in the successful treatment of chondrosarcoma involving the spine. 

Although en bloc resection can improve the clinical results of the surgical treatment of chondrosarcoma, the procedures do not come without complications. In a review of 134 patients who underwent en bloc resection at a single institution, complications were noted in 48 patients. A total of 43 major complications were noted in 27 patients, and 29 minor complications were noted in 28 patients. Major complications included vascular injuries to the vena cava and aorta, myocardial infarction, pulmonary embolism, deep infection requiring surgical debridement, transient renal failure, ureteral injury, temporary paraplegia, and an ex vacuo cerebral hematoma secondary to cerebrospinal fluid leak. Three patients died from complications related to the procedure [17]. 

## 6. Conclusion

Chondrosarcoma is a malignancy of bone that can present within the mobile spine and the sacrum. The effective staging of chondrosarcoma plays an essential role in the disease treatment. This family of tumors is known to be resistant to both chemotherapy and radiation therapy, thus treatment is directed toward surgery. The prognosis of chondrosarcoma involving the spine is related to the histological grade of the tumor, the age of the patient at the time of diagnosis, initial treatment taking place in a primary tumor center, and most importantly the adequacy of the surgical margins. Patients who undergo successful en bloc excision have recurrence rates as low as 20%.
